# Phosphoproteomics Reveals Selective Regulation of Signaling Pathways by Lysophosphatidic Acid Species in Macrophages

**DOI:** 10.3390/cells13100810

**Published:** 2024-05-09

**Authors:** Raimund Dietze, Witold Szymanski, Kaire Ojasalu, Florian Finkernagel, Andrea Nist, Thorsten Stiewe, Johannes Graumann, Rolf Müller

**Affiliations:** 1 Department of Translational Oncology, Center for Tumor Biology and Immunology, Philipps University, 35043 Marburg, Germany; dietze@staff.uni-marburg.de (R.D.); ojasaluk@staff.uni-marburg.de (K.O.); finkernagel@imt.uni-marburg.de (F.F.); 2 Institute of Translational Proteomics, Biochemical Pharmacological Centre, Philipps University, 35043 Marburg, Germany; 3 Core Facility Translational Proteomics, Philipps University, 35043 Marburg, Germany; 4 Bioinformatics Core Facility, Philipps University, 35043 Marburg, Germany; 5 Genomics Core Facility, Philipps University, 35043 Marburg, Germany; andrea.nist@imt.uni-marburg.de (A.N.); stiewe@uni-marburg.de (T.S.)

**Keywords:** lysophosphatidic acid, macrophages, phosphoproteomics, RHO/RAC1 signaling, cholesterol biosynthesis

## Abstract

Lysophosphatidic acid (LPA) species, prevalent in the tumor microenvironment (TME), adversely impact various cancers. In ovarian cancer, the 18:0 and 20:4 LPA species are selectively associated with shorter relapse-free survival, indicating distinct effects on cellular signaling networks. Macrophages represent a cell type of high relevance in the TME, but the impact of LPA on these cells remains obscure. Here, we uncovered distinct LPA-species-specific responses in human monocyte-derived macrophages through unbiased phosphoproteomics, with 87 and 161 phosphosites upregulated by 20:4 and 18:0 LPA, respectively, and only 24 shared sites. Specificity was even more pronounced for downregulated phosphosites (163 versus 5 sites). Considering the high levels 20:4 LPA in the TME and its selective association with poor survival, this finding may hold significant implications. Pathway analysis pinpointed RHO/RAC1 GTPase signaling as the predominantly impacted target, including AHRGEF and DOCK guanine exchange factors, ARHGAP GTPase activating proteins, and regulatory protein kinases. Consistent with these findings, exposure to 20:4 resulted in strong alterations to the actin filament network and a consequent enhancement of macrophage migration. Moreover, 20:4 LPA induced p38 phosphorylation, a response not mirrored by 18:0 LPA, whereas the pattern for AKT was reversed. Furthermore, RNA profiling identified genes involved in cholesterol/lipid metabolism as selective targets of 20:4 LPA. These findings imply that the two LPA species cooperatively regulate different pathways to support functions essential for pro-tumorigenic macrophages within the TME. These include cellular survival via AKT activation and migration through RHO/RAC1 and p38 signaling.

## 1. Introduction

Lysophosphatidic acid (LPA) was first identified as a significant tumor-promoting factor in the malignant ascites of ovarian carcinoma (OC) patients, where it was named “ovarian cancer activating factor” [1]. Ascites plays a critical role in the complex tumor microenvironment (TME) of OC, contributing to its peritoneal metastasis, immune suppression, and resistance to therapy. The detrimental impact of the OC TME is facilitated by a complex network of interactions between tumor cells, immune cells, and residential host cells [2,3,4,5,6]. At the heart of these interactions are multiple soluble mediators, acting as signaling molecules across different cell types and impinging on their functional properties. LPA is one of these mediators with profound effects on disease progression [7,8,9].

LPA encompasses a series of lipids, each structured with a glycerol core bound to a saturated or unsaturated fatty acid at the sn1 or sn2 positions, and a phosphate group at the sn3 position [10]. Common LPA species in plasma and ascites include those with 16:0, 18:0, 18:1, 18:2, and 20:4 fatty acids, connected through ester bonds (acyl-LPA) [11]. LPA is produced from phospholipids mainly via the action of a secretory phospholipase A and subsequent processing by the lysophospholipase D autotaxin [12,13]. LPA signaling is mediated by six G-protein-coupled receptors (LPAR1-6), activating a variety of signaling pathways. These include (i) Gα_12/13_ regulating RAC/RHO signaling, (ii) Gα_i/o_ modulating phospholipase C, (iii) phosphatidylinositol 3-kinase (PI3K), (iv) Gα_q/11_ signaling to phospholipase C, and (v) Gα_s_ stimulating adenylate cyclase [14,15]. LPAR1-3 form a receptor subgroup related to the endothelial differentiation gene (EDG) subfamily, while LPAR4-6 are structurally more similar to purinergic receptors [14,15].

The impact of LPA on cancer cell signal transduction, as well as its role in enhancing tumor cell migration and invasion are relatively well-established [4,9,16,17,18]. This is in marked contrast to its effect on macrophages, which, despite their crucial role in the OC TME, remains surprisingly understudied in a recent review [19]. Limited research has revealed the activation of the Pi3K pathway promoting macrophage survival [20,21,22] and the polarization towards a pro-inflammatory M1 phenotype [23,24]. There is also evidence of LPA influencing macrophage migration, yet results are mixed, showing both stimulatory and inhibitory outcomes [25,26,27]. If LPA species used are defined at all, a notable limitation of these studies is their focus on LPA 18:1, leaving species selectivity on signaling mechanisms unexplored. Specifically, the role of 20:4 LPA as the potentially most relevant species in OC [9] remains to be investigated. In the present study, we have addressed this gap through an unbiased phosphoproteomic approach comparing the effects of both 18:0 and 20:4 LPA on human monocyte-derived macrophages (MDMs).

## 2. Materials and Methods

### 2.1. Isolation and Culture of Monocyte-Derived Macrophages (MDMs) from Healthy Donors

Mononuclear cells from healthy adult donors isolated by Ficoll density gradient centrifugation from Leukoreduction System (LRS) chambers were kindly provided by the Center for Transfusion Medicine and Hemotherapy at UKGM. Materials were used with the informed consent of the donors and approved by the local ethics committee (205/10). Monocytes were seeded at approximately 2 × 10^7^ cells per 100 mm dish, 2 × 10^6^, 1 × 10^6^, or 0.5 × 10^6^ cells per well in 6-well, 12-well, or 24-well plates. The adherent cells were washed twice with 1 mL of phosphate-buffered saline (PBS) and differentiated for 6 days in RPMI1640 (Life Technologies, Darmstadt, Germany) supplemented with 5% human AB serum (Sigma Aldrich, Taufkirchen, Germany), 1 mM sodium pyruvate (Sigma Aldrich), and 20 ng/mL M-CSF. Under these conditions, cells expressing the macrophage markers CD206 and HLA-DR exceeded 95% by flow cytometry. Twenty-four hours prior to utilizing MDMs in experiments, the culture conditions were changed to a serum-free medium to mitigate interference from high levels of LPA in serum.

### 2.2. LPA and Recombinant Proteins

The LPA species 16:0 (857123P), 18:0 (857128P), and 18:1 (857130P) were obtained from Avanti Polar Lipids (Alabaster, AL, USA), 18:2 LPA (L-0182), 20:4 LPA (L-0204) from Echelon Bioscience (Salt Lake City, UT, USA), MCP-1/CCL2 (571402) and M-CSF (BLD-574804) from Biozol (Eching, Germany). LPA mix mirroring its composition in ascites contained the following components: 20% 16:0 LPA, 5% 18:0 LPA, 10% 18:1 LPA, 32.5% 18:2 LPA, and 32.5% 20:4 LPA [9].

### 2.3. Measurement of Intracellular cAMP

To quantitatively determine cyclic AMP (cAMP) levels in cell lysate, we utilized the cAMP Parameter Assay Kit (KGE002B, R&D Systems, Minneapolis, MN, USA) following the manufacturer’s protocol. Prior to stimulation with 5 µM LPA or solvent, cells were treated with the cAMP phosphodiesterase inhibitor IBMX (13347, Cayman Chemicals, Ann Arbor, MI, USA) at a concentration of 0.1 mM for 15 min. Optical density readings at 450 nm were obtained using a SpectraMax 340 microplate reader (MWG Biotech, Ebersberg, Germany) in technical duplicates with wavelength correction measured at 570 nm. cAMP concentrations were determined based on a standard curve generated using a four-parameter logistic (4-PL) curve-fit method.

### 2.4. RNA Sequencing 

MDMs were treated with 5 µm LPA species or solvent (ethanol) for 3 h and total RNA was isolated using the NucleoSpin RNA II kit (740955.250, Macherey-Nagel, Düren, Germany). RNA-Seq was performed and data were processed as described previously [28,29] using Ensembl 108 [30] and STAR 2.7.10a [31]. Statistical evaluations were performed with edgeR (4.0.0) [32]. Expression data were normalized to ‘counts per million’ (CPM) for visualization. RNA-Seq data were deposited at EBI ArrayExpress (accession number E-MTAB-13993). 

### 2.5. Sample Preparation for Mass Spectrometry (MS)

Differentiation of the MDMs was performed as described above. The cells were serum starved for 24 h prior to treatment with 5 µM of 18:0 LPA, 20:4 LPA, LPA mix, or solvent for 30 min. Cells were lysed with 4% SDS in 50 mM Tris-HCl buffer with Roche PhosSTOP (#4906845001; Sigma-Aldrich) and complete protease inhibitor cocktail (P8340, Sigma Aldrich). Cell extracts were subjected to proteomic profiling in quadruplicates at the Core Facility Translational Proteomics at Philipps University Marburg. Following protein content estimation using BCA (Dreieich, Germany), approximately 1 mg of protein was reduced and alkylated by the addition of DTT to a final concentration of 10 mM and incubation at 95 °C for 10 min, as well as iodoacetamide to a final concentration of 13 mM and incubation for 30 min at RT in the dark. Before digestion, samples were diluted with 50 mM triethylammonium bicarbonate TEAB buffer to a final SDS concentration of 0.5%. Trypsin (Promega, Madison, WI, USA) was added to a final 1:50 ratio of enzyme to protein and digestion was performed over-night, at 37 °C with shaking and was stopped by the addition of trifluoroacetic acid (TFA) to a final concentration of 1.5%. Precipitating SLS was removed by 10 min centrifugation. Peptides were purified using solid-phase extraction on C18 Sep-Pak, Vac-1cc-100 mg columns (Waters, Eschborn, Germany) according to the manufacturer’s instructions. Purified peptides were first dried, then resuspended in 400 μL of 50 mM TEAB. Peptide concentration was estimated using the Pierce Fluorimetric Peptide Assay. In total, 200 μg per sample per tandem mass tag (TMT) was taken for labelling and volumes were equalized with 50 mM TEAB buffer. 

Five separate TMT-mixes were designed, with each mix representing a separate donor with additional reference channel: TMT126—Solvent, TMT127—LPA 18:0, TMT128—LPA 20:4, TMT129—LPA mix, TMT130—reference). TMT labelling was performed according to instructions. Following sample mixing, the volume was reduced by half using evaporation to remove acetonitrile. Trifluoroacetic acid was added to a final concentration of 0.5%. TMT-mixes were purified using solid-phase extraction on C18 Sep-Pak, Vac-1cc-100 mg columns (Waters, Ire-land) according to the manufacturer’s instructions. The eluate was split into two: approximately 10% for downstream proteome analysis and approximately 90% for subsequent phospho enrichment. Both aliquots were evaporated to dryness.

Phosphorylated peptides were enriched using the High-SelectTM Fe-NTA Phosphopeptide Enrichment Kit (Catalog Number A32992, Thermo Scientific) according to the manufacturer’s instructions. Both phospho- and proteome aliquots were subsequently fractionated using the Pierce™ High pH Reversed-Phase Peptide Fractionation Kit (Catalog number: 84868, Thermo Scientific). The entire procedure resulted in the generation of 80 MS samples: 5 × 8 protein and 5 × 8 phospho fractions per TMT mix. Dried fractionated peptides were resuspended in 100 μL 0.1% formic acid (FA) prior to LC-MS analysis. For proteome fractions, 4 μL (approximately 500 ng of peptides) was injected. For phospho fractions, 10 μL was used for injection.

### 2.6. Mass Spectrometry (MS)

Peptides were analyzed by liquid chromatography tandem mass spectrometry (LC/MS2) on an Exploris 480 instrument connected to an Ultimate 3000 rapid separation liquid chromatography (RSLC) nano instrument and a nanospray flex ion source (all Thermo Fisher Scientific, Waltham, MA, USA). Peptide separation used a reverse-phase high-performance liquid chromatography (HPLC) column (75 μm by 42 cm) packed in-house with C18 resin (2.4 μm; Dr. Maisch HPLC GmbH, Ammerbuch, Germany). The peptides were first loaded onto a C18 precolumn (preconcentration set-up) and then eluted in the backflush mode with a gradient from 98% solvent A (0.15% formic acid) and 2% solvent B (99.85% acetonitrile and 0.15% formic acid) to 25% solvent B over 48 min, continuing from 25 to 35% of solvent B for another 20 min. The flow rate was set to 300 nL/min. Data were acquired in a data-dependent mode (DDA) using one high-resolution MS scan at a resolution of 60,000 (*m*/*z* 200) with a scan range of 320–1650 *m*/*z*, followed by DDA scans limited to a 2 s cycle time, with the first mass set to 199 *m*/*z* at a resolution of 45,000. Detailed settings are uploaded with the mass spectrometric raw data to the ProteomeXchange Consortium with dataset identifier: PXD051172, via the MassIVE partner repository (https://massive.ucsd.edu/, MassIVE ID: MSV000094459).

Peptide spectrum matching was performed using MaxQuant (version 2.0.3.0) against the Human UniProt database (March 2022) with TMT quantification and reporter ion distribiution correction (uploaded in the repository). The output was filtered to a 1% false discovery rate on both peptide and protein levels, tryptic cleavage following K*, R*, as well as a maximum of 2 missed cleavages. Cysteine carbamidomethylation was included as a fixed modification, while methionine oxidation, asparagine, and glutamine deamidation, as well as serine, threonine, and tyrosine phosphorylation were set as variable modifications. The full list of settings may be found in the “mqpar.xml” file uploaded to the ProteomeXchange repository.

The MaxQuant output table “Phospho (STY)Sites.txt” was imported into Python (Jupyter version 6.1.4). Identified phosphosites were filtered for control/reverse decoys, transformed with log2(x + 1) for variance stabilization and subjected to replicate-set paired *t* tests followed by Benjamin–Hochberg adjustment to estimate false discovery rates.

### 2.7. Immunoblotting and Quantification

Immunoblotting was performed according to standard protocols using the following antibodies: p-p38 T180/Y182 (4511, Cell Signaling, Frankfurt, Germany), p38 (9228, Cell Signaling #9228), pAKT S473 (4060, Cell signaling), AKT (2920, Cell signaling), p-MLC T18/S19 (3674, Cell Signaling), MLC2 (sc-517244, Santa Cruz Biotechnology, Heidelberg, Germany). Imaging and quantification were carried out using ChemiDoc MP and Image Lab software version 5 (Bio-Rad, Hercules, CA, USA). Phosphoform signals were normalized against the respective total protein signals. 

### 2.8. Staining of Actin Filaments

MDMs were differentiated on glass coverslips as described above. For actin filament staining, the cells were incubated with 5 µM LPA species for 30 min, fixed with 4% paraformaldehyde for 10 min at room temperature, and permeabilized with 0.3% Triton X100 for 5 min. Actin filaments were stained with Phalloidin-California Red Conjugate (1:1000; AAT Bioquest, Sunnyvale, CA, USA) for 30 min at room temperature. Glass coverslips were mounted on microscope slides using mounting medium with DAPI (VEC-H-1200, Vector, Burlingame, CA, USA) and sealed with nail polish. Images were taken at 40× magnification and processed on a widefield microscope (Leica DM5500, Leica Microsystems, Wetzlar, Germany). Images of three random fields per sample were analyzed with imageJ 1.54 (https://imagej.net/ij/).

### 2.9. Transwell Migration Assay

Macrophages were differentiated as described above. Cells were gently scraped off the culture dish and, after centrifugation, 2 × 10^5^ cells were transferred into the transwell insert containing 400 µL serum-free medium with solvent or 5 µM LPA. The bottom compartment contained 500 µL full culture medium with 50 ng/mL recombinant MCP-1/CCL2. After 24 h, the migrated cells were fixed with 4% paraformaldehyde and stained with 1% *w*/*v* crystal violet in 20% methanol/H_2_O. The stained membranes were washed three times with H_2_O, dried, and mounted on microscopy slides with VECTASHIELD Antifade Mounting Medium (Biozol). Migrated cells were analyzed on a Leica DMBI3000 microscope at 10× magnification and counting of three random fields per sample.

### 2.10. Macropinocytosis Assay

MDMs were differentiated in 6-well plates and pretreated with 5 µM LPA or solvent (ethanol) for 30 min. Macropinocytic activity was assessed 60 min subsequent to the addition of FITC-Dextran (70 kDa, 0.5 mg/mL). Following gentle detachment of cells from the culture plates, samples were analyzed via flow cytometry. Gating was performed with cells preincubated for 60 min on ice to distinguish FITC-Dextran binding to the cell surface from FITC-Dextran uptake as described [33].

### 2.11. Functional Annotations

Proteomic data were functionally annotated by Reactome pathway analysis [34] using the online tool of the Gene Ontology Resource website at http://geneontology.org. For enrichment analyses of biological functions [35], we used the “Biological Process Complete” function of Gene Ontology Ressource [36,37] at https://geneontology.org. Pathway analysis of transcriptome data was performed by overrepresentation analysis using the ConsensusPathDB (CPDB) database [38,39].

### 2.12. Other Statistical Analyses

Comparative data were statistically analyzed by a paired Student’s *t*-test (two-sided, equal variance). Significance levels are indicated as ****, ***, **, and * for *p* < 0.0001, *p* < 0.001, *p* < 0.01, and *p* < 0.05, respectively.

## 3. Results

### 3.1. Phosphoproteomics Identifies LPA-Species-Selective Target Proteins in MDMs

**Figure 1:**
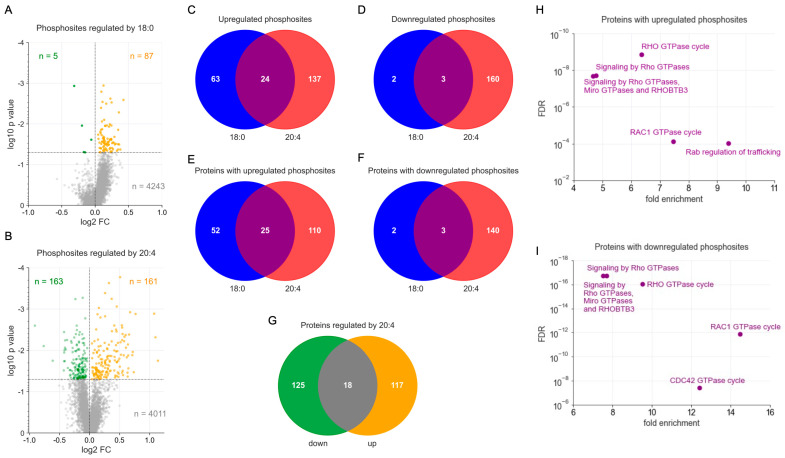
Phosphoproteomics of 18:0 and 20:4 LPA-treated MDMs. Serum-deprived MDMs were treated with 5 µM LPA or solvent for 30 min prior to MS-based phosphoproteomic analysis. (**A**) Volcano plot of 18:0 LPA regulated phosphosites. Green: downregulated sites (nominal *p* < 0.05). Orange: upregulated sites (*p* < 0.05). Grey: sites not significantly affected. Horizontal line: significance threshold (*p* = 0.05). Numbers: significantly downregulated (green) and upregulated (orange) genes. (**B**) Volcano plot for 20:4 LPA regulated phosphosites analogous to panel (**A**). (**C**) Venn diagram of 18:0 and 20:4 LPA upregulated phosphosites (nominal *p* < 0.05). (**D**) Venn diagram of 18:0 and 20:4 LPA downregulated phosphosites (*p* < 0.05). (**E**) Venn diagram of proteins with phosphosites (in panel (**C**)) up- and/or downregulated by 18:0 LPA. (**F**) Venn diagram of proteins with phosphosites (in panel (**D**)) up- and/or downregulated by 20:4 LPA. (**G**) Venn diagram showing the overlap (gray area; n = 18) of proteins with both up- and downregulated phosphosites for 20:4 LPA (shown in panels (**E**,**F**). (**H**) Reactome analysis of proteins with phosphosites upregulated by 18:0 LPA and/or 20:4. The plot shows the most significant (FDR) enriched non-redundant terms. (**I**) Reactome analysis as in panel (**H**) for proteins with downregulated phosphosites.

To decipher the LPA-regulated signaling network in macrophages with a focus on identification of potential LPA-species-specific effects, we analyzed MDMs treated with 18:0 LPA, 20:4 LPA, or solvent control for 30 min using an unbiased MS-based phosphoproteomic screen. This comprehensive analysis revealed n = 4335 phosphorylation sites assignable to annotated genes (refer to Table S1). After normalization against the corresponding protein group signals, we identified n = 87 and n = 161 phosphorylation sites upregulated (nominal *p* < 0.05) after treatment with 18:0 LPA and 20:4 LPA, respectively, as compared to mock treatment, with an intersection of n = 24 sites (Figure 1A–C). Conversely, n = 5 and n = 163 phosphorylation sites were found downregulated as compared to mock treatment for 18:0 and 20:4 LPA treatments, respectively, with 3 sites common to both (Figure 1A,B,D). Mapping upregulated phosphorylation sites onto protein groups yielded n = 187 proteins with assignable gene names, with n = 25 shared by the two LPA species (Figure 1E), while downregulated phosphorylation sites mapped to n = 145 proteins, with an intersection of only n = 3 (Figure 1F). These findings indicate that 20:4 LPA exerts a stronger influence on the phosphoproteome of MDMs as compared to the 18:0 variant. Considering the strong association of 20:4 LPA with ovarian cancer (OC) survival [9], these observations underscore the critical importance of this LPA species within the TME.

### 3.2. Functional Annotation of 18:0 and 20:4 LPAs Target Proteins

**Figure 2:**
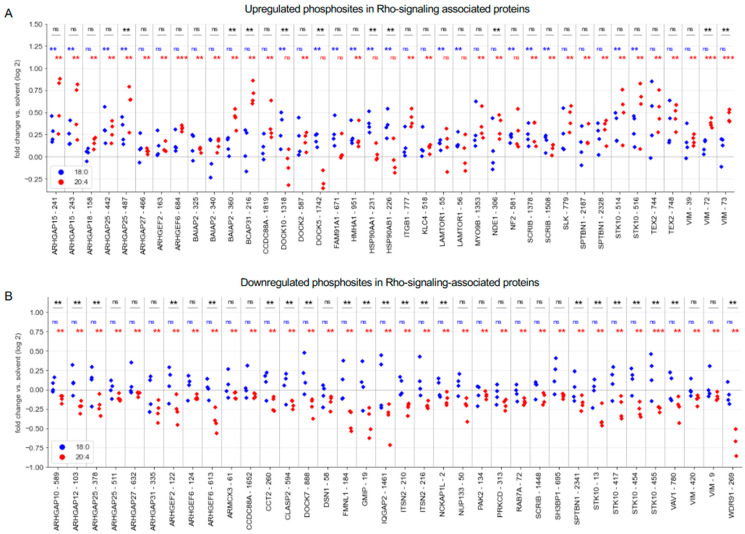
RHO/RAC1-signaling-associated phosphosites regulated by LPA species in MDMs. Phosphoproteomic data from Figure 1 for individual proteins and samples. (**A**) Rho signaling associated proteins with upregulated phosphosites by 18:0 LPA (blue) or 20:4 LPA (red) identified by phosphoproteomics (nominal *p* < 0.05; FC > 1.1). The plot shows the FC for n = 4 biological replicates. (**B**) Plot as in panel (**A**) for proteins with downregulated phosphosites. *** *p* < 0.001; ** *p* < 0.01 for AA versus solvent by paired *t* test. ns, not significant.

The 20:4 LPA species affected n = 260 proteins in total, among which n = 18 contained both up- and downregulated phosphorylation sites (Figure 1G). This suggests an intricate regulatory network of both stimulatory and inhibitory phosphorylation events acting on the same protein. This notion is supported by Reactome-based annotation [40] of the combined 18:0/20:4 LPA-regulated proteins (Figure 1H,I), which identified similar pathways for proteins with up- or downregulated phosphorylation sites, i.e., signal transduction mechanisms centered around RHO/RAC1 GTPases. Very similar results were obtained when 18:0 and 20:4 LPA-regulated proteins were analyzed separately (Table S2; 18:0 LPA-downregulated proteins were not analyzed due to lack of coverage). The sets of 18:0 and 20:4 LPA-regulated proteins involved in RHO/RAC1 signaling overlapped but were not identical (Figure 2; Table S1), indicating functional cooperation by the two LPA species.

The identified proteins include regulators and targets of RHO/RAC1 pathways [41], i.e., guanine exchange factors (ARHGEF and DOCK family members), GTPase activating proteins (ARHGAPs), and the protein kinases PAK2 and STK10 (upregulated phosphorylation sites in Figure 2A; downregulated sites in Figure 2B). Consistent with these findings, functional annotation by enrichment analysis [37] using the Gene Ontology Resource [39] pinpointed biological functions linked to regulation of cytoskeleton organization as the most significantly impacted. This was evident for proteins with upregulated phosphosites, notably in “positive regulation of cell motility” (enrichment 5.0-fold; FDR = 0.04) as well as for proteins with downregulated phosphosites in “positive regulation of cell motility” (FDR = 4.5 × 10^−7^), Collectively, our phosphoproteomic analysis reveals a significant impact of 20:4 LPA, and to a lesser extent of 18:0 LPA, on various signaling pathways, especially those related to reorganization of the actin filament network. 

### 3.3. Phosphoproteomic Analysis of an Ascites-Emulating Mixture of LPA Species 

**Figure 3:**
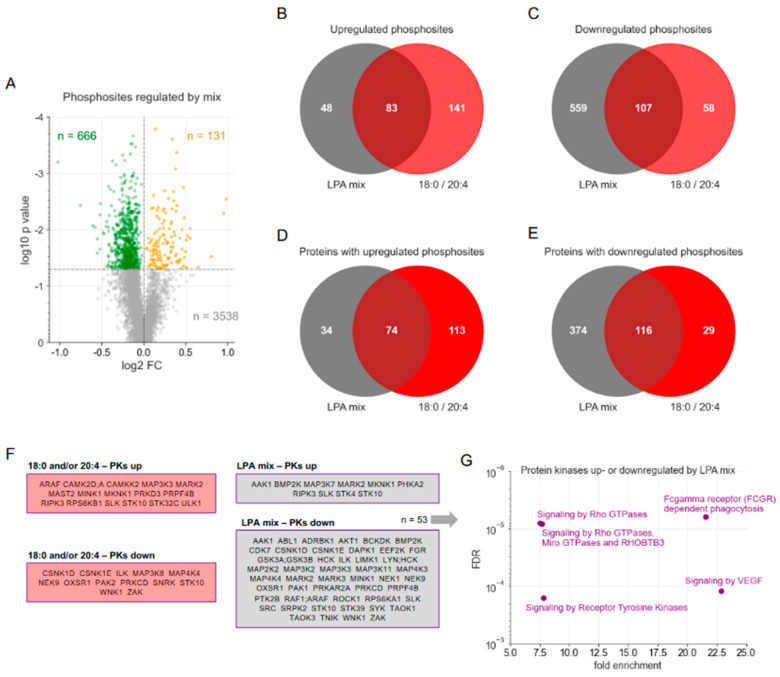
Comparative phosphoproteomic analysis of MDMs treated with 20:4 LPA versus ascites-emulating LPA mix. MDMs were treated as in Figure 1. (**A**) Volcano plot of LPA-mix regulated phosphosites. Green: downregulated sites (nominal *p* < 0.05). Orange: upregulated sites (*p* < 0.05). Grey: sites not significantly affected. Horizontal line: significance threshold (*p* = 0.05). Numbers: significantly downregulated (green) and upregulated (orange) genes. (**B**) Venn diagram of phosphosites upregulated by LPA mix or 18:0/20:4 LPA species (nominal *p* < 0.05). (**C**) Venn diagram of phosphosites downregulated by LPA mix or 18:0/20:4 LPA species (*p* < 0.05). (**D**) Venn diagram of proteins with phosphosites (in panel (**B**)) upregulated by LPA mix or 18:0/20:4 LPA species. (**E**) Venn diagram of proteins with phosphosites (in panel (**B**)) downregulated by LPA mix or 18:0/20:4 LPA species. (**F**) Protein kinases with phosphosites up- or downregulated by LPA mix (gray area) or 18:0/20:4 LPA species (pink area). (**G**) Reactome analysis of LPA-mix-regulated protein kinases. The plot shows the most significant (FDR) enriched non-redundant terms.

To evaluate the ascites-equivalent joint impact of LPA species on macrophages, we further analyzed the phosphoproteome of MDMs subjected to a mixture of LPA species, mirroring their proportional representation in OC ascites [9], subsequently referred to as LPA mix. The volcano plot presented in Figure 3A illustrates that after normalization against associated protein group IDs, n = 131 phosphorylation sites were upregulated (nominal *p* < 0.05) by the LPA mix, while n = 666 sites were downregulated (see Table S1 for the complete dataset). As shown by the Venn diagrams in Figure 3B,C, n = 83 (63.4%) of the n = 131 upregulated sites and n = 107 (16.1%) of the n = 666 downregulated sites, respectively, overlapped with those affected by 18:0 and/or 20:4 LPA. The phosphosites upregulated by the LPA mix were found in n = 108 proteins, with n = 74 (68.5%) of these shared with the union of the separate 18:0/20:4 LPA treatments (Figure 3D). Phosphosites downregulated by the LPA mix were observed in n = 490 proteins, with n = 116 (23.7%) of these in common with 18:0 and/or 20:4 LPA treatment (Figure 3D). These findings underscore the significant role of the 18:0/20:4 LPA species in modulating the LPA-impacted phosphoproteome in MDMs, particularly regarding upregulated sites. Nonetheless, it is apparent that other LPA species, not individually analyzed here, also play a substantial role, especially in relation to the downregulated sites. The data further show that a number of sites are regulated by 18:0 and/or 20:4 LPA but not by the LPA mix. We attribute this to the higher concentration of the individual LPA species (5 µM) tested as compared to their concentration in LPA mix (5 µM total LPA with 0.25 µM and 1.625 µM 18:0 and 20:4 LPA, respectively).

The conclusions drawn from these analyses gain further support by the data in Figure 3F, which demonstrates that 8 out of 10 protein kinases with sites upregulated by the LPA mix overlap with those influenced by 20:4 LPA. Conversely, only 9 out of 48 (18.8%) protein kinases with downregulated sites were common between 20:4 LPA and the LPA mix. Additional evidence is provided by the functional annotation depicted in Figure 3G, highlighting RHO-GTPase-regulated signaling as a prominent pathway. Notably, the most significant finding for the LPA mix was “Fcγ receptor-dependent phagocytosis”, which was not identified in the functional annotation of 18:0/20:4-LPA-regulated proteins (Figure 1H,I), indicating that this function might be mediated by other LPA species not individually examined in this study.

The secondary messenger cAMP is induced by LPA through G_s_-interacting receptors [15,42]. To explore this pathway, we measured the concentration of intracellular cAMP following the stimulation of MDMs with 18:0 LPA, 20:4 LPA, LPA mix, or solvent control. As illustrated in Figure S1, these treatments did not result in significant changes in cAMP levels, suggesting that cAMP-dependent signal transduction may play a minimal, if any, role in mediating LPA signals in this cell type.

### 3.4. LPA-Species-Selective Phosphorylation of MLC2, AKT, and p38 in MDMs

**Figure 4:**
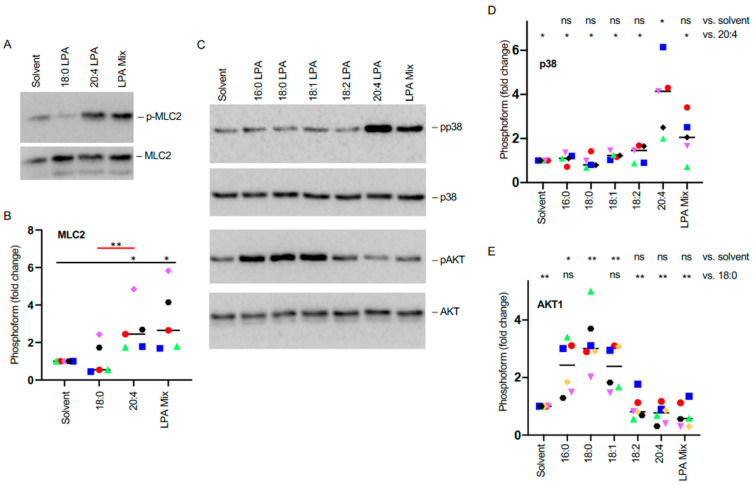
Regulation of protein kinase phosphorylation by LPA in MDMs. Serum-deprived MDMs were treated with 5 µM LPA or solvent for 30 min and analyzed by immunoblotting. (**A**) Characteristic immunoblot showing the effect of 18:0 LPA, 20:4 LPA, and LPA mix on MLC2 phosphorylation. MLC was included for normalization of phosphoform signals. (**B**) Quantification of n = 5 biological samples (FC relative to solvent). (**C**) Immunoblot as in panel (**A**) analyzing effects of 18:0 LPA, 20:4 LPA and LPA mix on AKT1 and p38 phosphorylation. (**D**) Quantification of changes in pp38 levels for n = 5 biological replicates for 5 different LPA species. (**E**) Quantification of changes in pAKT1 levels for n = 5 biological replicates. Colored data points represent individual samples, the horizontal line indicates the median. Significance was tested by paired *t*-test: ** *p* < 0.01; * *p* < 0.05, ns, not significant.

Motivated by the regulation of cytoskeletal dynamics suggested by the analyses above, we subsequently broadened our investigation to phosphosites of potential relevance not identified in the MS analysis. Specifically, we analyzed the phosphorylation of myosin light chain 2 (MLC2) at T18/S19, AKT at S473 and p38 at T180/Y182 in response to 18:0 and 20:4 LPA. MLC plays a crucial role in cellular functions that are dependent on actomyosin-driven contraction, such as cell motility [43]. AKT and p38 are pivotal in numerous biological functions, including cell survival and migration, underscoring their importance in signaling pathways [20,44,45]. Immunoblotting demonstrated that the phosphorylation of MLC2 at T18/S19 (Figure 4A,B) and of p38 at T180/Y182 (Figure 4C,D) were significantly elevated in response to 20:4 LPA and LPA mix, but were not significantly affected by the 18:0 variant. Conversely, AKT phosphorylation at S473 was triggered by 18:0 LPA and not by 20:4 LPA or LPA mix (Figure 4C,D). Changes in AKT1 phosphorylation were also observed in the phosphoproteome regulated by the LPA mix (Figure 3F; Table S1). However, in this instance, LPA led to a decrease in AKT1 phosphorylation at S124, indicating the existence of multiple regulatory mechanisms acting on AKT1. We attribute the observed differences between MS-based phosphoproteomics and immunoblotting to distinct detection thresholds inherent to these methods.

When including further LPA species in our analysis, we observed no substantial influence of 16:0, 18:0, 18:1, and 18:2 LPA on p38 phosphorylation. In contrast, both 16:0 and 18:1 LPA were found to enhance AKT1 phosphorylation, similarly to the 18:0 form, while 18:2 and 20:4 LPA had no significant impact (Figure 4D,E). These observations reinforce the concept of LPA-species-selective effects on the phosphoproteome of MDMs, including both common actions (16:0, 18:0, and 18:1 LPA) and distinct responses (20:4 LPA). 

### 3.5. Effect of LPA Species on the Actin Filament Network and Migration of MDMs

**Figure 5:**
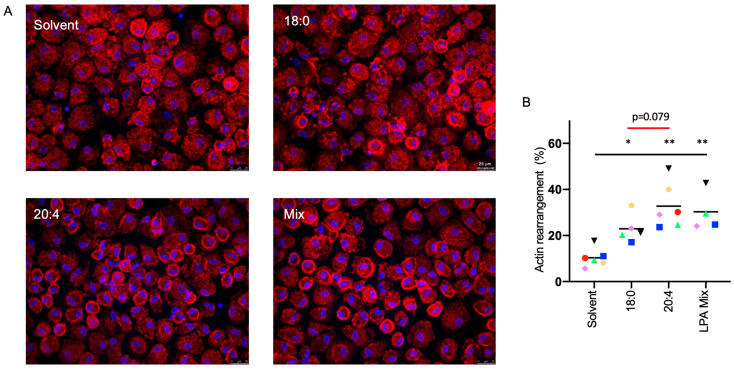
Actin cytoskeleton rearrangement in MDMs treated with 18:0 of 20:4 LPA. (**A**) Representative photomicrographs of phalloidin-stained MDMs treated with 5 µM LPA species or solvent for 30 min. Nuclei were counterstained with DAPI. (**B**) Quantification of n = 4–6 biological replicates, 3 random fields were evaluated for each sample. Cells with distinct actin rearrangement (accumulation at the plasma membrane) were counted as positive. Colored data points represent individual samples, the horizontal line indicates the median. Significance was tested by paired *t*-test: ** *p* < 0.01; * *p* < 0.05.

In light of the significant influence of LPA on RHO/RAC1 signaling pathways, we extended our investigation to assess its impact on RHO/RAC1-associated biological functions. Phalloidin staining demonstrated a marked and significant reorganization of actin filaments induced by 20:4 LPA, apparent from their accumulation at the plasma membrane (Figure 5A). Quantitative analysis showed that both 20:4 LPA and the LPA mix produced a similar effect, while 18:0 LPA resulted in a weaker, yet still significant, rearrangement (Figure 5B).

**Figure 6:**
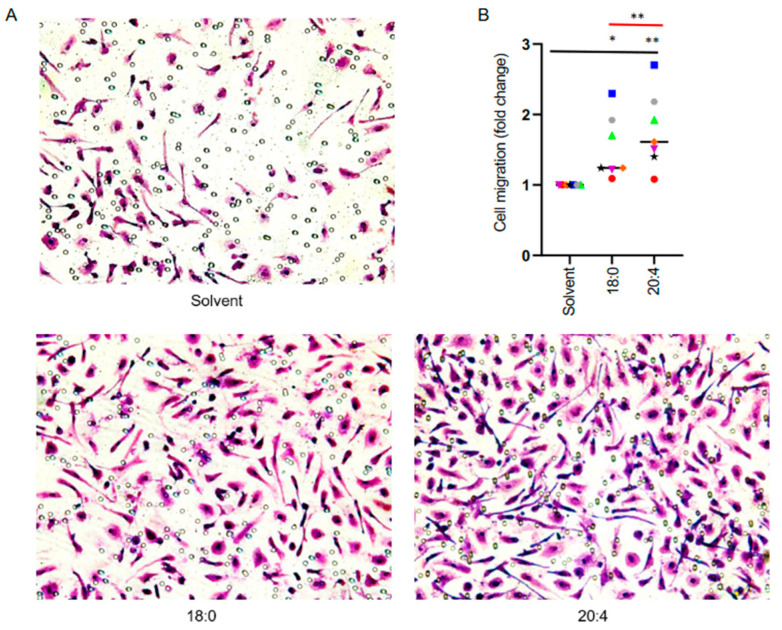
Transwell migration assay of MDMs treated with 18:0 of 20:4 LPA. (**A**) Crystal-violet-stained after 24 h migration towards a gradient of human serum and MCP-1/CCl-2 (50 ng/mL) in the presence of 5 µM LPA species or solvent. (**B**) Quantification of n = 7 biological replicates, 3 random fields were analyzed for each sample. Colored data points represent individual samples, the horizontal line indicates the median. Significance was tested by paired *t*-test: ** *p* < 0.01; * *p* < 0.05.

Given the critical functions of RHO/RAC1 signaling and actomyosin contraction in cell motility [41,46], we further studied potential effects of both LPA species on the directed migration of macrophages towards a gradient of serum and MCP-1/CCL2 (chemotaxis). The results of transwell migration assays shown in Figure 6 revealed a significant stimulatory effect by both 18:0 and 20:4 LPA, with the 20:4 form emerging as the more potent mediator, consistent with the data obtained by phalloidin staining. 

Collectively, the results of these functional assays validate our phosphoproteomic analyses and emphasize the dominant role of the 20:4 species in biological processes linked to actin dynamics.

### 3.6. Transcriptional Response of MDMs to 18:0 of 20:4 LPA

**Figure 7:**
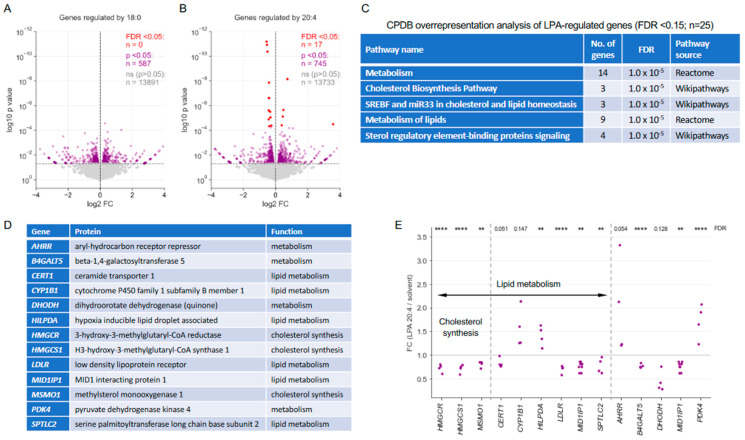
Transcriptional profiling of MDMs treated with 18:0 of 20:4 LPA. Serum-deprived MDMs were treated with 5 µM LPA or solvent for 3 h prior to RNA-Seq analysis. (**A**) Volcano plot depicting genes significantly regulated by 18:0 LPA (purple: nominal *p* < 0.05; red: FDR > 0.05) or showing no significant change (grey). (**B**) Volcano plot of genes regulated by 20:4 LPA. (**C**) CPDB overrepresentation analysis of 20:4 LPA-regulated genes (FDR < 0.15; n = 25). The table shows the top 5 hits. (**D**) Functions of the metabolism-associated genes identified by the analysis in panel (**C**). (**E**) Data points for individual metabolism-associated genes (as in (**D**)) and n = 4 samples. The plot shows FC values (20:4 LPA/solvent) based on RNA-Seq data. **** FDR < 0.0001; ** FDR < 0.01 by paired *t* test and Benjamini–Hochberg correction across all genes analyzed by RNA-Seq. FDR values > 0.05 are indicated as numbers.

Finally, we aimed at determining whether the selective effects observed for different LPA species extend to transcriptional regulation. To this end, we determined the transcriptome of MDMs treated with 18:0 LPA, 20:4 LPA or solvent for 3 h. RNA-Seq revealed a relatively muted transcriptional response to both LPA species (low FC, low number of significantly regulated genes). Among the genes affected, n = 587 were regulated (nominal *p* > 0.05) by 18:0 LPA and n = 745 by 20:4 LPA, with only n = 14 and n = 21 genes, respectively, exhibiting an FC greater than 3 (refer to Figure 7A; Table S3). Notably, none of the genes influenced by 18:0 LPA achieved a false discovery rate (FDR) below 0.15, whereas n = 25 genes responsive to the 20:4 LPA species did, n = 17 of these with an FDR < 0.05 (Figure 7A,B; Table S3). 

Intriguingly, CPDB overrepresentation analysis of genes regulated by 20:4 LPA (FDR < 0.15) revealed a pronounced and significant association with metabolic pathways, notably those related to cholesterol biosynthesis and other lipid-associated processes (Figure 7C,D). Remarkably, three genes with critical functions in cholesterol biosynthesis [47,48], i.e., *HMGCR*, *HMGCS1*, and *MSMO1*, exhibited uniform downregulation across all samples (Figure 7E. These observations suggest that LPA suppresses the expression of key enzymes in both the mevalonate pathway and postsqualene biosynthesis [47,48], which is selective for the 20:4 species.

## 4. Discussion

Our phosphoproteomic analysis pinpointed RHO/RAC1-dependent signal transduction as the principal pathway affected by 18:0 LPA, 20:4 LPA or a combination of LPA species simulating the conditions in OC ascites. Although many proteins and phosphorylation sites were found altered under all conditions examined, we also discovered specific sites uniquely impacted by 18:0 LPA, 20:4 LPA, or other LPA species present in the LPA mix, indicating a cooperative interplay among different LPA species. RHO/RAC1 signaling plays a critical role in regulating actin filaments and the cytoskeleton [41,46], and thus in LPA-regulated actomyosin-dependent biological processes such as cell movement and migration. The present study highlights these roles in MDMs and corroborates findings from prior research on other cell types [14,49,50].

In macrophages, RHO/RAC1 signaling and its impact on the actin cytoskeleton also play a crucial role in phagocytosis [51]. We were, however, unable to detect significant LPA-related effects in a macropinocytosis assay, which monitored the uptake of dextran by MDMs (see Figure S3). Nonetheless, LPA may influence other forms of phagocytosis that operate through mechanisms distinct from macropinocytosis [17,18,19,52,53].

Our data also indicate that 20:4 LPA affects the expression of multiple genes coding for proteins with critical roles in lipid metabolism. Of particular interest may be the coordinate downregulation of three enzymes with key roles in sterol/cholesterol biosynthesis. These are (i) 3-hydroxy-3-methylglutaryl-CoA reductase, which catalyzes the critical step of HMG-CoA formation within the mevalonate pathway; (ii) HMG-CoA synthase 1, a key rate-controlling enzyme of the mevalonate pathway; and (iii) methylsterol monooxygenase 1, pivotal in the postsqualene segment of cholesterol biosynthesis [47,48]. These observations suggest that LPA suppresses both the mevalonate pathway and postsqualene biosynthesis, indicating an inhibitory effect of LPA on the de novo synthesis of cholesterol and other isoprenoids. 

Lipid metabolic reprogramming, encompassing processes such as fatty acid uptake, intracellular storage, catabolism, as well as altered cholesterol metabolism, significantly influences the polarization of TAMs, consequently impacting tumor progression [54]. Notably, the promotion of pro-tumorigenic TAM polarization is correlated with cholesterol depletion, primarily stemming from reduced cholesterol biosynthesis and enhanced efflux [55,56]. Hence, it is plausible that LPA plays a pivotal role in this context, as our data revealed the suppression by LPA of multiple genes essential for cholesterol synthesis. 

The mevalonate pathway has been correlated with macrophage polarization, with most studies indicating increased mevalonate metabolism associated with the classical M1 state [57,58]. Consistent with these observations, increased cholesterol efflux has been reported to promote anti-inflammatory reprogramming, including an inhibition of IFNγ functions [55]. However, contradictory findings have been reported [59]. Inhibiting the mevalonate pathway reduces geranylgeranyl pyrophosphate synthesis, leading to impaired protein geranylgeranylation, decreased PI3K activation, and consequently a proinflammatory state. These seemingly conflicting results may align with the mixed M1/M2 polarization observed in TAMs in OC ascites [60].

LPA species exhibit diverse biological activities that are influenced by the nature of their fatty acid side chains, especially when comparing saturated with unsaturated fatty acids [50]. In the context of OC, for example, it was reported that urokinase plasminogen activator secretion by tumor cell lines was stimulated by 18:1 LPA, but not by 18:0 LPA or 16:0 LPA [61]. This variation in biological activity may be attributed to the differential affinities LPARs display towards various LPA species [62]. This leads to the question whether the selective effects of LPA species observed in our phosphoproteomic, transcriptomic and functional analyses could stem from the activation of specific receptors.

The EDG family members LPAR1 (EDG2) and LPAR2 (EDG4) exhibit broad ligand specificity for LPA species with either unsaturated or saturated fatty acids in thee sn1 or sn2 position, alongside differences among 14:0, 16:0, and 12:0 [63]. For LPAR3 (EDG7) the highest reactivity was observed for ∆9-unsaturated fatty acids (18:1, 18:2, and 18:3 LPA) followed by 16:1 LPA, 20:4 LPA, and LPA species with saturated fatty acids [63]. Likewise, non-EDG LPARs have a preference for LPA species with unsaturated fatty acids, i.e., LPAR4/P2Y9: 18:1 > 18:0 > 16:0 > 14:0 [64]; LPAR5/GPR92: 18:1 > LPA 20:4 = LPA 16:0 = LPA 18:3 > LPA 18:0 > LPA 20:0 [65]; and LPAR6/P2Y5: 18:2 > 18:1 > 20:4 > 18:0 > 16:0 > 14:0 [66].

In the present study, we exclusively used acyl-LPA species with fatty acids at the sn1 position containing <10% of sn2-acyl-LPAs, as specified by the manufacturer. As reported by Bandoh (2000), both sn1-18:0 and sn1-20:4 LPA exhibit low affinity towards LPAR3. In contrast, both LPA species have a high affinity for LPAR1 and LPAR2. Coupled with the considerably lower expression of LPAR3 in MDMs compared to LPAR1 and LPAR2 (Figure S2), these findings may imply that LPAR1 and/or LPAR2 rather than LPAR3 play a dominant role in mediating LPA-triggered effects. However, as LPAR1 and LPAR2 have very similar affinities for sn1-18:0 and sn1-20:4 LPA [63], differential binding properties cannot explain their selective effects observed in our studies.

In contrast to the EDG-type LPAR receptors, LPAR6 exhibits a higher affinity for binding with 20:4 LPA as compared to 18:0 LPA [66]. An inherent characteristic of this receptor is its ability to induce cAMP synthesis [15,42]. However, our investigation revealed no significant impact on intracellular cAMP levels with either 18:0 LPA, 20:4 LPA, or LPA mix (Figure S1). Consequently, it is unlikely that LPAR6 solely mediates the selectivity for 20:4 LPA in MDMs. Other surface receptors, such as the nuclear receptor peroxisome proliferator activated receptor γ [67], have also been reported to bind LPA, potentially contributing to its effects. Moreover, it remains uncertain whether the binding affinities reported in vitro or in other cell types accurately reflect the properties of LPAR receptors in macrophages. It is plausible that interacting molecules or protein modifications could exert cell-type-specific influences on receptor functionality. This is exemplified by free fatty acid receptor 4 (FFA4), which can functionally desensitize LPAR1 [68]. While these questions lie beyond the immediate scope of our study, they warrant future investigation in the context of LPA signaling in OC.

## Data Availability

Phosphoproteomics data were uploaded to the ProteomeXchange repositorybvwith dataset identifier: PXD051172. RNA-Seq data were deposited at EBI ArrayExpress (accession no. E-MTAB-13993).

## Supplementary Materials

The following supporting information can be downloaded at: https://www.mdpi.com/article/10.3390/cells13100810/s1. Table S1: Phosphoproteomics data. Table S2: Reactome analyses. Table S3: RNA-Seq data. Figure S1: cAMP assay of LPA-treated MDMs. Figure S2: RNA-Seq data for *LPAR* expression in MDMs. Figure S3: Flow cytometric analysis of macropinocytosis by LPA-treated MDMs.

## Abbreviations

CPDB: ConsensusPathDB; CPM: counts per million; EDG: endothelial differentiation gene; FC: fold change; FDR: false discovery rate; GEF: guanine nucleotide exchange factor; HPLC: high-performance liquid chromatography; LPA: lysophosphatidic acid; LPAR: lysophosphatidic acid receptor; MDM: monocyte-derived macrophage; MLC2: myosin light chain 2; MS: mass spectrometry; OC: ovarian cancer; RNA-Seq: RNA sequencing; RSLC: rapid separation liquid chromatography; TMT: tandem mass tag; TEAB: triethylammonium bicarbonate; TFA: trifluoroacetic acid; TME: tumor microenvironment.
