# Supplementary material for: Phosphoproteomics Reveals Selective Regulation of Signaling Pathways by Lysophosphatidic Acid Species in Macrophages
Source: Cells. 2024 May 9;13(10):810. doi: 10.3390/cells13100810 (PMC11119170; doi:10.3390/cells13100810)
Supplement: Supplementary file 1 [file cells-13-00810-s001.zip › Supplemental Figures.pdf]

Supplemental Figures

Phosphoproteomics reveals selective regulation of signaling pathways by lysophosphatidic acid species in macrophages

Raimund Dietze[1], Witold Szymanski[2,3], Aditya Bhagwat[2,3], Kaire Ojasalu[1], Florian Finkernagel[1,4], Andrea Nist[5], Thorsten Stiewe[5], Johannes Graumann[2,3] and Rolf Müller[1]*

[1]Department of Translational Oncology, Center for Tumor Biology and Immunology, Philipps University, Marburg, Germany

[2]Institute of Translational Proteomics, Biochemical Pharmacological Centre, Philipps University, Marburg, Germany

[3]Translational Proteomics Core Facility, Philipps University, Marburg, Germany

[4]Bioinformatics Core Facility, Philipps University, Marburg, Germany

[5]Genomics Core Facility, Philipps University, Marburg, Germany

*Author to whom correspondence should be addressed.

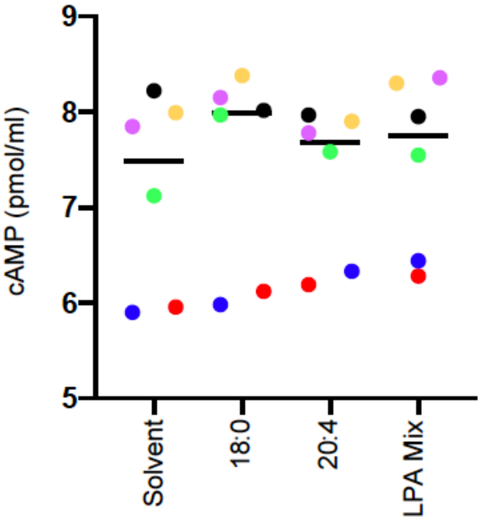

Figure S1: Concentration of cAMP in MDM lysates after pstimulation with 5 µM LPA or solvent (EtOH) for 15 min measured by competitive enzyme immunoassay. Each dot represents a biological replicate (n=5). Horizontal lines represent the median. The p values obtained by two-sided, paired t-test indicate non-significance across all treatments compared to the solvent control.

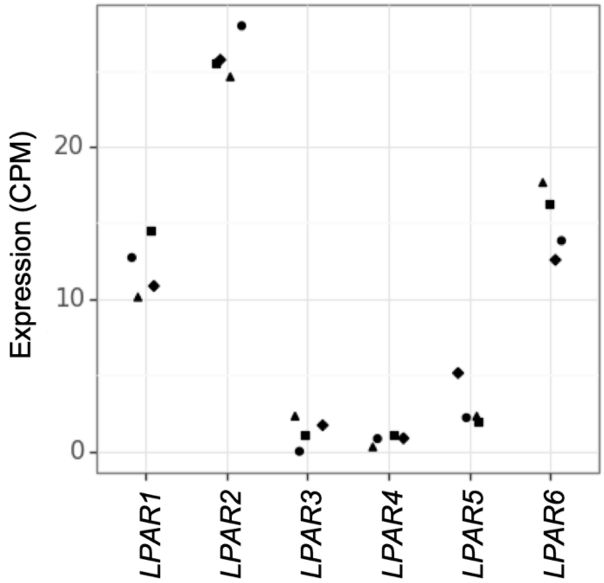

Figure S2: Expression of *LPAR* genes in non-polarized (M0) MDMs determined by RNA-Seq. Each symbol represents a biological replicate (n=4 donors).

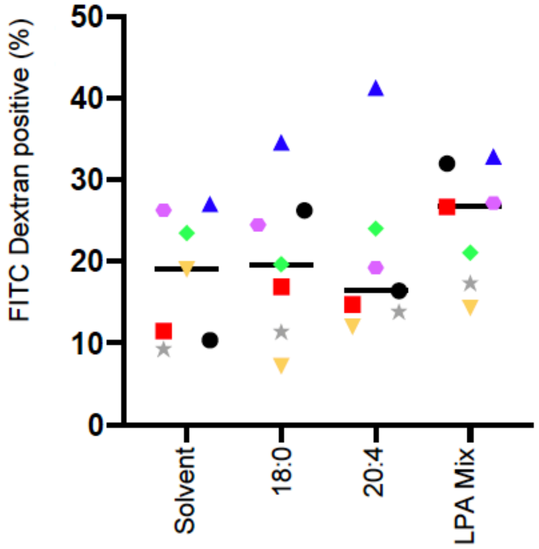

Figure S3: Flow cytometric analysis of FITC-Dextran macropinocytosis by MDMs treated with 5 µM LPA or solvent for 24 h. Untreated MDMs incubated on ice to invoke a complete inhibition of pinocytosis were used as negative control for gating. Each symbol represents a biological replicate (n=7). Horizontal lines represent the median. The p values obtained by two-sided, paired t-test indicate non-significance across all treatments compared to the solvent control. .
